# Antineoplastic activity of biogenic silver and gold nanoparticles to combat leukemia: Beginning a new era in cancer theragnostic

**DOI:** 10.1016/j.btre.2022.e00714

**Published:** 2022-02-26

**Authors:** Ebrahim Mostafavi, Atefeh Zarepour, Hamed Barabadi, Ali Zarrabi, Linh B. Truong, David Medina-Cruz

**Affiliations:** a Stanford Cardiovascular Institute, Stanford University School of Medicine, Stanford, CA 94305, USA; b Department of Medicine, Stanford University School of Medicine, Stanford, CA 94305, USA; c Department of Biomedical Engineering, Faculty of Engineering and Natural Sciences, Istinye University, Sariyer, Istanbul 34396, Turkey; d Department of Pharmaceutical Biotechnology, School of Pharmacy, Shahid Beheshti University of Medical Sciences, Tehran, Iran; e Department of Chemical Engineering, Northeastern University, Boston, MA 02115, USA

**Keywords:** Biosynthesis, Metallic nanomaterials, Gold NPss, Silver NPs, Therapeutic, Leukemia

## Abstract

The American Cancer Society estimated around 61,090 new cases of leukemia were diagnosed, and around 23,660 people died from this disease in the United States alone in 2021. Due to its burden on society, there is an unmet need to explore innovative approaches to overcome leukemia. Among different strategies that have been explored, nanotechnology appears to be a promising and effective approach for therapeutics. Specifically, biogenic silver and gold nanoparticles (NPs) have attracted significant attention for their antineoplastic activity toward leukemia cancer cells due to their unique physicochemical properties. Indeed, these nanostructures have emerged as useful approaches in anti-leukemic applications, either as carriers to enhance drug bioavailability and its targeted delivery to a specific organ or as a novel therapeutic agent. This review explores recent advances in green synthesized nanomaterials and their potential use against leukemia, especially focusing on silver (Ag) and gold (Au) nanostructures. In detail, we have reviewed various eco-friendly methods of bio-synthesized NPs, their analytical properties, and toxicity effects against leukemic models. This overview confirms the satisfactory potency of biogenic NPs toward leukemic cells and desirable safety profiles against human native cells, which opens a promising door toward commercializing these types of nontherapeutic agents if challenges involve clinical validations, reproducibility, and scalability could be resolved.

## Graphical abstract

**Figure:**
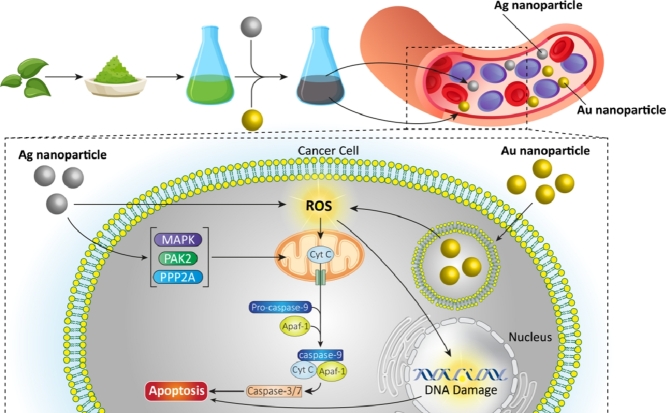

## Acronyms

nanoparticles: (NPs)
silver: (Ag)
gold: (Au)
acute lymphocytic leukemia: (ALL)
acute myeloid leukemia: (AML)
chronic lymphocytic leukemia: (CLL)
chronic myeloid leukemia: (CML)
gold nanoparticles: (AuNPs)
silver nanoparticles: (AgNPs)
reactive oxygen species: (ROS)
doxorubicin: (DOX)
half minimal inhibitory concentrations: (IC50s)
Acridine orange: (AO)
ethidium bromide: (EtBr)
ferulic acid: (FA)
sinapic acid: (SA)
caffeic acid: (CA)
chlorogenic acid: (CHA)
interleukin 1β: (IL-1β)
Human umbilical vein endothelial cells: (HUVEC)
Lens culinaris: (L. culinaris)

## 1 Introduction

### 1.1 Cancer and leukemia

Cancer is known as a group of diseases related to the abnormal growth of cells in the form of tumors that can either remain into the original place of formation, giving it the name of primary cancer, or spread into other nearby tissues and form new ones, known as metastatic or secondary cancer [1]. This process involves a wide set of different up-regulation and down-regulation of cellular genes and proteins such as those that control cell growth and death, cell-adhesion receptors or receptors related to cell motility, genes related to tumor suppression, and several others [2]. Although cancer triggers vastly depend on the type of disease, general causes have been established, including environmental and genetic factors, hormones, diet, or even infectious agents [3]. Nowadays, cancer is known as one of the third main causes of death globally, leading to about 10 million deaths in 2020. Statistical data showed that about 19.3 million new cancer cases were detected in 2020 [4] and it is expected that those numbers will grow up to 30 million by 2040 [5]. Clearly, despite great advances in developing new technologies for diagnosis and treatments, cancer is still a large burden on society.

Leukemia is a type of cancer resulting from non-controlled cell proliferation in the tissues which are responsible for the fabrication of blood cells and includes the lymphatic system and bone marrow. Leukemia usually impacts the normal functioning of white blood cells that have the role of fighting against infections [6]. In normal conditions, these cells have orderly growth and division based on the body's needs, while in the case of leukemia, high amounts of abnormal white blood cells with functional defects are produced by the bone marrow. To efficiently cure leukemia, first, sensitive and accurate diagnostic tools are essential, which can be achieved by functionalized NPs as biosensors [7], CRISPR/Cas9 systems [8], DNA conjugation [9], and ultra-resolution imaging equipment, etc. Segmentation of leukemia types is usually due to the wide variety of areas in which the disease can emerge [10]. Leukemia is specifically classified according to its cell types (lymphocytes and myeloid cells) and the duration of their development (acute disease (with a rapid development) and chronic disease (with a slow rate)) [11], [12], [13]. As such, it is easy to distinguish between acute lymphocytic leukemia (ALL); and acute myeloid leukemia (AML); both types have chances to become chronical and categorized as chronic lymphocytic leukemia (CLL); and chronic myeloid leukemia (CML). Moreover, each of these types has different subcategorizes [14, 15].

**Fig. 1:**
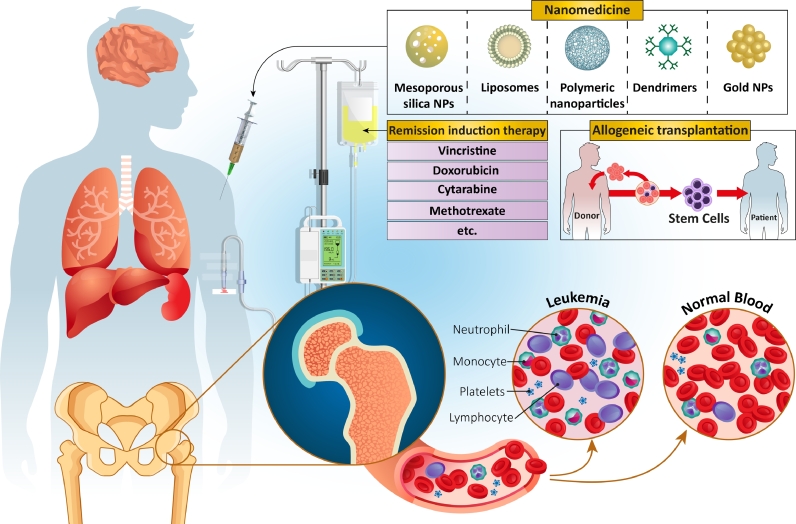
Some of the different methods used for Leukemia treatment.

Unfortunately, leukemia treatment is complex due to its heterogeneous nature that leads to using therapeutic methods directed according to the phenotype, genotype, and risk (Fig. 1). In recent years, an interesting approach has been developing antileukemic drugs by employing agents that act on specific molecular targets or pathways that can regulate leukemic cell death, cycle progression, epigenetic alteration, and signal transduction [16]. The Ubiquitin-proteasome system is one of those attractive targets [16, 17]. On the other hand, short-term intensive chemotherapy is a common treatment for most types of cancer, has been isolated to only leukemia patients with disorders in their B-cell [18]. Nevertheless, a typical treatment for leukemia is included three main steps; a remission-induction therapy, which is continued by the intensification (or consolidation) therapy, and finally a continuation treatment for eliminating the residual of leukemia [19]. Indeed, specific treatment is used for the central nervous system with different lengths of time, related to the systemic treatment intensity, recurrence risk, and whether or not cranial irradiation is applied.

**Fig. 2:**
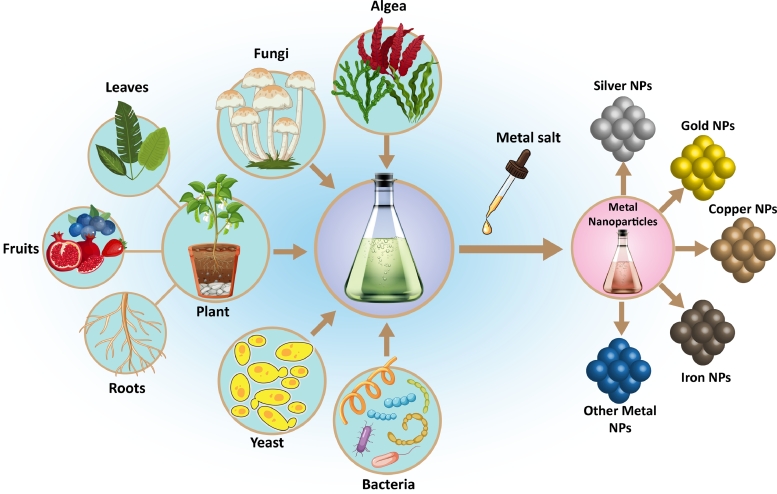
Different sources used for green synthesize of metal NPs. Microorganisms, different parts of plants, fungi, and algae are the biological sources that can be applied for the fabrication of several types of metal nanoparticles.

**Fig. 3:**
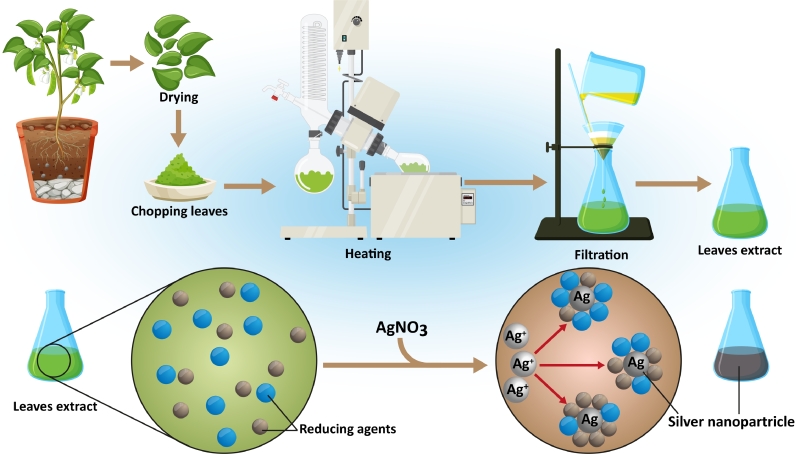
Schematic of plant extraction and biosynthesis NPs fabrication. Plant extracts are collected at first via a heating process. These extracts, which are contained different reducing agents, are used for the biofabrication of nanoparticles.

**Fig. 4:**
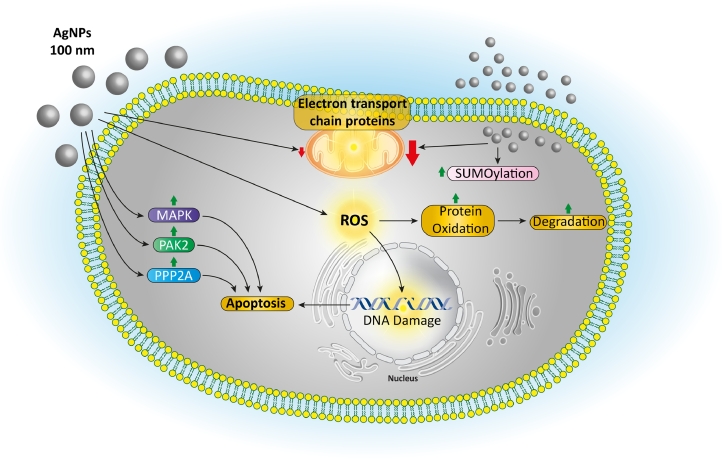
Different cytotoxicity mechanisms of AgNPs against cancer cells. These nanoparticles could increase the production of reactive oxygen species (ROS), leading to protein degradation and DNA damage. Moreover, they could enhance apoptosis via stimulating different intracellular pathways.

**Fig. 5:**
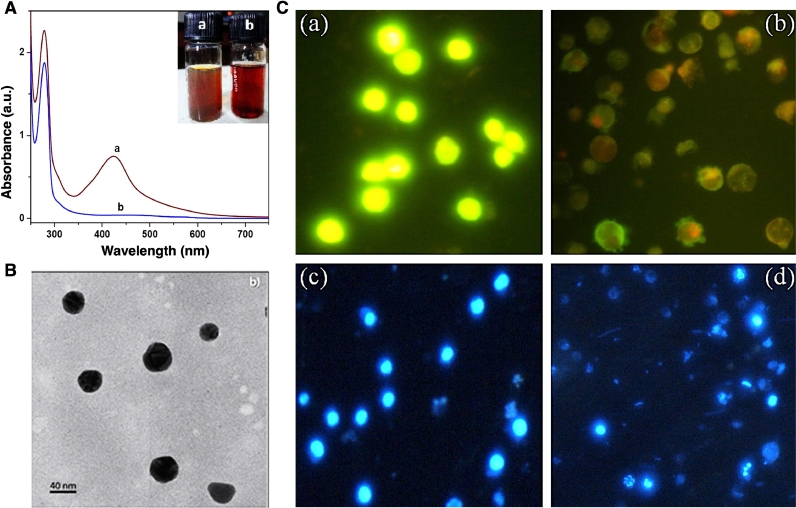
(A) UV-Visible analysis of Butea monosperma bark extract solution a) with and b) without AgNPs. (B) HRTEM analysis of the biosynthesized AgNPs. (C) Fluorescence images of KG-1A cells stained with AO-EtBr (a and b) and DAPI (c and d), before (a and c) and after (b and d) exposed with AgNPs (images are taken at 400x magnification). Reprinted from [86] with permission from Elsevier.

**Fig. 6:**
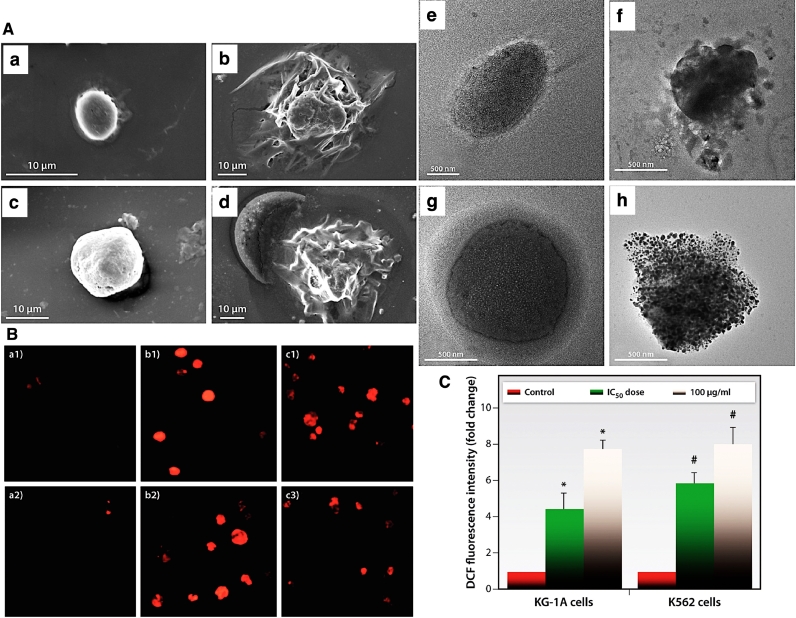
(A) Cell membrane structure observation by SEM imaging (a-d, scale bars= 10 µm), a& c showing untreated KG-1a & K-562 cells, while b & d showing AgNPs treated KG-1A & K-562 cells, and their corresponding (e-h) TEM images (scale bars= 500 nm), respectively. (B) Comparison of nucleus structure of control cells (a1 and a2) and K-562 cancer cells before (b1-c1) after (b2-c2) exposing with two concentrations (IC50 (b) and 100 µg/ml (c)) of green synthesized AgNPs. (C) Production of ROS inside the KG-1A and K562 Leukemia cancer cells in response to different concentrations of biosynthesized AgNPs. Reprinted from [90].

**Fig. 7:**
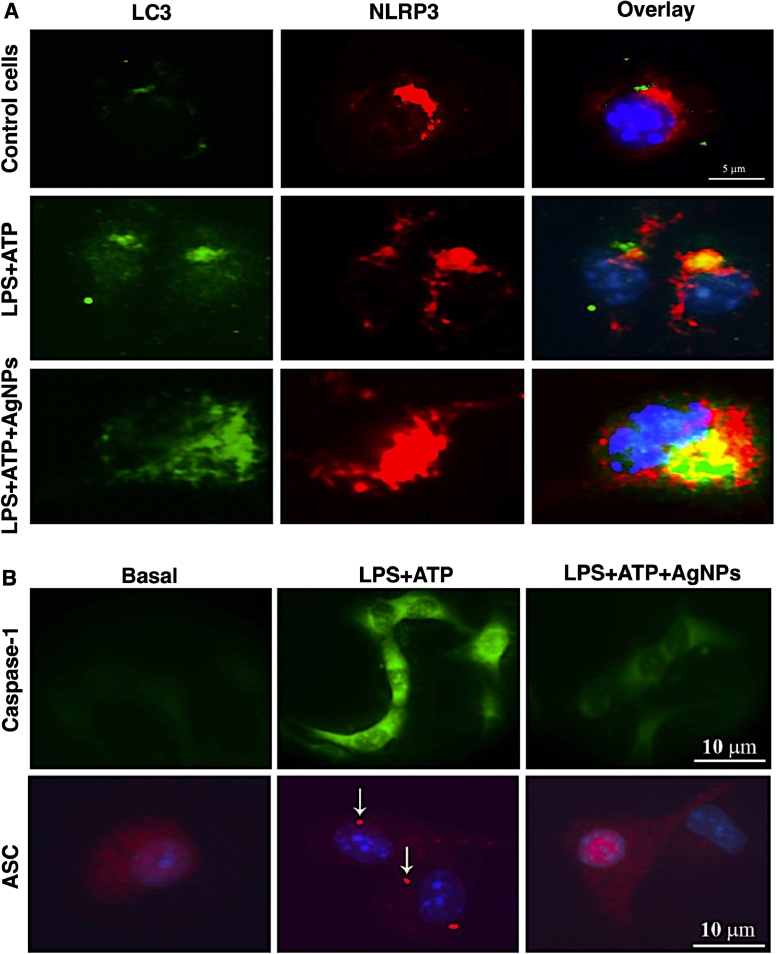
Elevation of autophagy via targeting (A) NLRP3 and (B) Casoase-1 and ASC, by green synthesized AgNPs. Reprinted from [93].

**Fig. 8:**
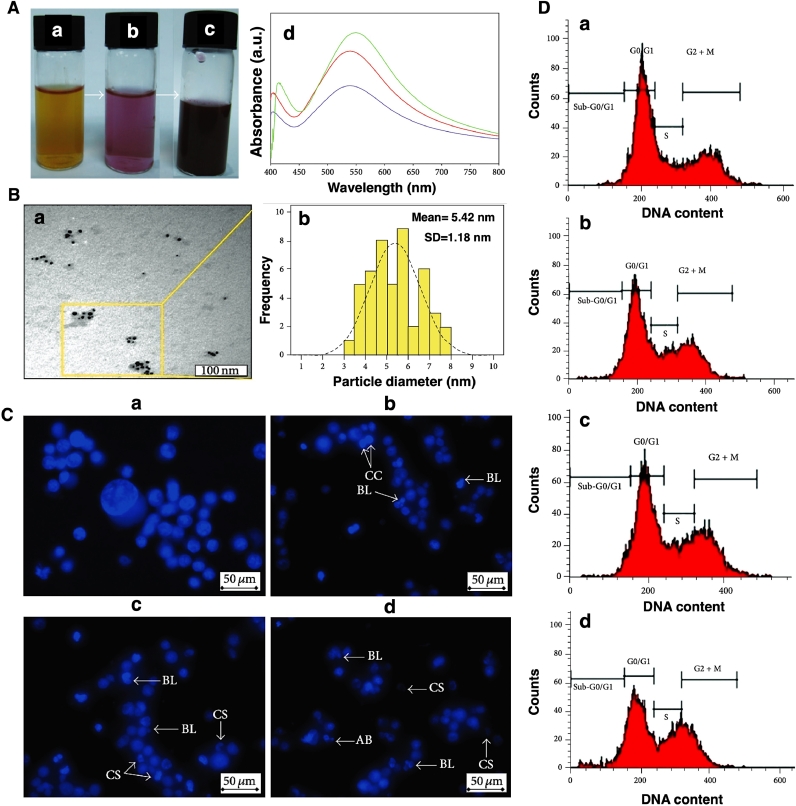
(A) Bio-synthesis of AuNPs in the presence of the aqueous extract of Sargassum muticum, (a-c) preparation of NPs during the time, (d) UV-Visible spectra of the prepared NPs. (B) (a)TEM image and (b) size distribution graph of the as-prepared NPs. (C) Fluorescent images of K562 cells stained by Hoechst 33,342, a) control cells with normal size and morphology, and cells treated by AuNPs after b) 24 h, c) 48 h, and d) 72 h. The morphology of the cells changed and apoptotic bodies appeared by increasing the time. (D) Flow cytometry results of K562 cells before (a) and after (b) 6 h, (c) 12, and 24 h (d) treatment with AuNPs. Reprinted from [107] with permission from Hindawi.

**Fig. 9:**
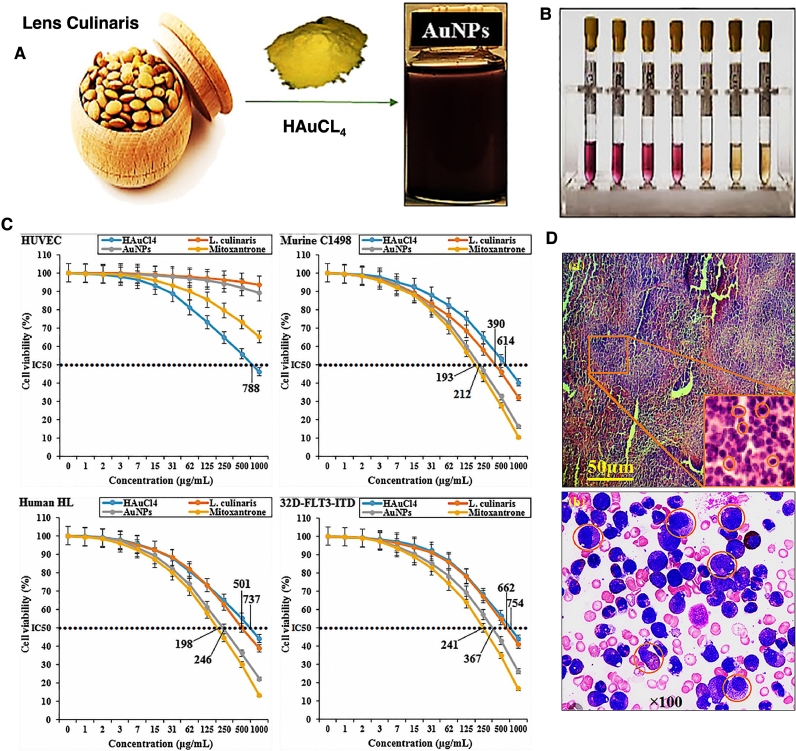
(A) Biosynthesis of AuNPs from L. culinaris extract. (B) Antioxidant activity of the prepared NPs. (C) Cytotoxicity activity of HAuCl4, L. culinaris, AuPs, and mitoxantrone against HUVEC, Murine C1498, Human HL-60/vcr, and 32DFLT3-ITD cell lines. (D) *In vivo* results of anticancer activity of AuNPs, a) The leukemic blasts infiltrating in histological stain of the spleen, and b) Leukocytosis in the smear of peripheral blood. Reprinted from [114] with permission from Wiley.

Remission-induction therapy is another therapeutic method used for leukemia treatment with the aim of destroying nearly all of the initial burden leukemia cells and is based on the administration of a steroid hormone, such as glucocorticoid, chemotherapy medications, such as vincristine, and at least one other chemical agent. At least four different types of drugs are normally used to treat nearly all young adults and (very)-high-risk ALL children in remission-induction therapy.

Also, several attempts have been made to improve the effectiveness of induction therapy by considering the fact that the prevention of drug resistance and improving therapeutic rate could be obtained via rapid and complete elimination of burden leukemia-cells [20]. After restoring normal hematopoiesis (formation of blood cellular components) in patients, they will be exposed to the next step of the treatment process, intensification therapy [21]. The normal therapeutic regimens used in this section include co-administration of high-dose methotrexate and mercaptopurine, and then high-dose of asparaginase used for an extended period. In the final step, re-induction treatment is used, which is necessary to reach a successful ALL treatment and is based on repeated administration of the initial induction therapy used during the first few remission months [22].

Immune-based methods are one of the newest therapeutic methods considered for leukemia treatment. In this case, the same as other types of cancer, different methods can be used, including conjugation of anti-body to drugs, inhibition of the programmed cell death protein 1 (PD1), T cell immunoglobulin, and cytotoxic T lymphocyte antigen 4 (CTLA4), via immune checkpoint blockers, and finally vaccines. However, these therapeutic methods are in their early stages, and several studies are needed to reach the decisive results [23, 24].

One other therapeutic method is allogeneic transplantation, which is based on transplanting new cells taken from cord blood or from a matched unrelated donor. This treatment can increase the chances of survival -among adults with ALL to about 45–75% compared to patients who use chemotherapy (30–40%) [25, 26]. This treatment method has some survival limitations (near 35%) after transplantation of allogeneic stem cells resulting from toxicity related to the therapy, specifically due to the infections and graft-versus-host disease. Therefore, it is critical to optimize the risk stratification, reduce treatment toxicity, adapt therapeutic protocols, and increase supportive care [27].Most of the treatments mentioned above, although widely used, are not free of drawbacks for the patient. For instance, low blood cell counts are often considered as a side-effect. In other words, while the AML could reduce the amounts of normal blood cells production from one side, chemotherapy could also affect and destroy these normal blood cells from the other side. This could lead to the generation of different types of blood cells deficiency, including anemia (related to red cells), thrombocytopenia (related to the platelets), neutropenia, and monocytopenia (related to the white cells) [28]. Another relevant complication in leukemia treatments is the risk of infection. During AML treatment and in the presence of chemotherapy, the probability of the infections is significantly increased due to the deficiency in the performance of neutrophils and monocytes. The bacterial and fungal infections could even enter the blood due to mouth and intestinal lining damage [29].

In the case of allogeneic stem cell transplantation, there is also a high risk of generating graft *versus* host diseases that are developed due to the activation of the donor's immune cells against the normal cells of patients [30]. One other type of chemotherapy side-effect is tumor lysis syndrome, which happens when the chemotherapy induction phase is applied to treat patients with a large number of leukemic cells. In other words, the destruction of the leukemia cells leads to the breaking of their membrane, and their contents are released to the blood, which tends to the changes in the chemical composition of blood that could damage different organs of the body [31].

Consequently, there is an increasing need for alternative solutions and treatment methods that deliver a better therapeutic effect while minimizing the side-effects of others and being complicated for the patient. Research is moving towards novel anti-leukemia therapies, in which one of the most interesting and most recent approaches is the application of different types of nanosized materials for treating leukemia, known as nanomedicine, which will be described in detail in the following section [32], [33], [34].

### 1.2 Nanomedicine as a novel approach to combat leukemia

The emerging field of nanotechnology has allowed for dramatic improvements in the field of medicine [35]. There is substantial evidence supporting the use of nanoparticles (NPs) to improve selectivity and to serve as an enhanced drug delivery vehicle upon current platforms [36]. Additionally, the ability of nanomaterials to manipulate properties such as tiny size, targeting ability, high loading capacity, and sustain released pattern provides easier control over the behaviors of drugs once they enter the patient body, which in turn results in higher selectivity and bioavailability of drug at the targeted site along with reducing adverse side-effects [37]. Because of their obvious advantages, nanomedicines have been implemented in approved cancer therapies as early as the 1990s.

Therapeutic applications of nanomedicine show a progression over time from pegylated liposomes as drug delivery vehicles for anticancer therapy as early as 2010 [38] to using NPs as useful anticancer therapies themselves. In the case of leukemia, two important nano-formulations have been generated for the proper delivery of chemotherapeutic drugs (mitoxantrone and flavopiridol) to the targeted cells in leukemia patients. Utilizing NPs has shown that the nanoscale can help in increasing the circulation half-life and bioavailability of the drugs, reducing their renal clearance, and decreasing the normal tissue exposure with high concentrations of drug for both of these drugs [39]. Indeed, different liposomal formulations containing mitoxantrone have been widely investigated for use in humans [40]. Flavopiridol is the other type of chemotherapeutic drug whose clinical usage is restricted due to its affinity for attachment with the plasma proteins along with its low water solubility [41]. Accordingly, utilizing nanoformulations could enhance the solubility of this drug, act as a barrier for its interaction with different plasma proteins, and eliminate the toxicity effect of flavopiridol formulations on non-targeted cells [42, 43].

Nanomaterials not only could provide more selectivity and reduce the adverse side-effects, but they can also be used as means for transporting drugs across the BBB [44]. Thus, there are reasons to believe that moving forward with nanomedicine as anticancer therapies has real-world applications and benefits that will exceed the confines of a research lab setting in the near future, especially in the treatment of leukemia. This transition is especially evident and potent when considering metallic nanostructures, which have demonstrated significant antibacterial efficacy in the past, and are beginning to be examined for their anticancer properties [45].

It is mentioned in some literature that, while they can synergistically increase the efficacy of other drug products, nanometals themselves display cytotoxic effects that inhibit cancer cell growth selectively [46]. For instance, there has been explicit expeditionary work on *in vitro* cytotoxicity assays of leukemia cell lines exposed to gold nanoparticles (AuNPs), demonstrating their anticancer effect and cytocompatible properties for cancer and healthy cells, respectively [47]. This evidence provides insights into nanometals as promising tools that could improve traditional chemotherapies' performance and reduce their potentially harmful side effects.

Still, the use of NPs is not free of drawbacks, and one of the most important factors associated with the potential complications of their use is how they are made, either by employing traditional physicochemical methods or using green nanotechnology-based platforms [48, 49]. Traditional synthesis methods comprise physicochemical approaches such as sol-gel, precipitation, laser ablation, and lithographic methods, among others. These methods allow for a straightforward, facile, and quick synthesis of nanomaterials with controllable parameters and a high degree of versatility. However, the downside is related to the release of potentially toxic by-products, the use of harsh reaction conditions, and the need for extra steps after synthesis for purification and functionalization of the nanostructures and to avoid aggregation or toxicology problems [50, 51]. On the other hand, green nanotechnology-based methods offer a way to overcome most of these disadvantages, as explained below.

### 1.3 Green nanotechnology in the treatment of cancer

Green nanotechnology is a sub-class of green chemistry, the approach of material synthesis using existing mechanisms in nature. Specifically, green nanotechnology refers to the formation of NPs from various sources of organisms, including algae, yeast, plants, bacteria, fungi, and viruses (Fig. 2) [52], [53], [54], [55]. In contrast with traditional physical and chemical synthesis methods, green synthesis does not produce toxic byproducts, and it doesn't need specialized equipment and materials, thus providing an eco-friendly method with increasing the overall scalability of the process. In addition, green NPs also show some superior properties compared to physicochemical methods, including ease of production, high biocompatibility, lack of toxic solvent, and being cost-effective [56]. Biosynthesis of NPs is a type of bottom-up approach based on the oxidation/reduction of inorganic ions into a nanosized system in the presence of microbial enzymes or plant phytochemicals that have the antioxidant ability or reducing capability [57].

Within the field of leukemia, these applications have started to attract attention, with most of the effort focusing on the use of biogenic silver (Ag) and gold (Au) nanostructures. Recently, the anticancer properties of biogenic NPs have been studied, and promising results have been obtained [58, 59]. In the following parts of this overview, we have described the recent works detailing the different biogenic methods of producing AgNPs and AuNPs and their subsequent evaluation of anticancer activities toward leukemia indication.

## 2 Green-synthesized metallic nanoparticles for the treatment of leukemia

The main mechanism of green synthesis of metallic NPs for leukemia indication has been based on the employment of plants’ extracts as both stabilizing and reducing agents. The reducing ability of plant extracts in the production of metal particles from their ions was referred to in the 1900s; however, the mechanism of these reducing agents was not well understood. Due to its simplicity, the application of live plants or the extract of the whole plant and plant tissue as reducing agents for the production of metal NPs has attracted significant attention during the last 30-years [60]. Approaches for producing NPs via utilizing plant extracts have several advantages compared to chemical ones. These are easy, scalable, and cost-effective methods [61] which are based on the bioreduction reaction mediated by a plant extract at room temperature and in the aqueous mixture of plant extract and the metal salt [62].

Plant extracts are full of biologically active molecules, including but not limited to proteins, polyphenols, and carbohydrates [63]. Secondary biomolecules, such as carotenoids, flavonoids, glucosinolates, and phytoestrogens, have also been found partially associated with the surface of generated metal NPs [64]. Since different extracts have various types of reducing agents with different concentrations, they could affect the properties of resulting NPs [65]. The general mechanism can be understood as a simple chemical reduction and further stabilization. Briefly, the reducing agent will start transforming the ions into small nuclei following an Ostwald ripening-like process, and eventually, the stabilizing agent will coat the nanostructures and stop the growth, thus stabilizing the final formulation [66]. Interesting to mention that most of the presented biomolecules could be applied as both reducing and stabilizing agents and always form a part of the final product, either as a nanocomposite or as a coated nanomaterial (Fig. 3) [67, 68].

### 2.1 Biogenic silver nanoparticles (AgNPs) for leukemia therapy

Among the diverse forms of biosynthesized metallic and metal oxide NPs, silver nanoparticles (AgNPs) have been demonstrated to be one of the most effective antimicrobial and anticancer agents due to their extraordinary properties [69], [70], [71], [72], [73], [74]. The most prominent outcomes of exposing cells with AgNPs are included affecting cellular metabolic activity via arresting the respiratory chain of the cells, influencing cell division through membrane inflicting damage, and inducing numerous subordinate effects, such as the production of reactive oxygen species (ROS), gene alteration, enhancement of apoptosis or necrosis, and elevating the oxidative stress and mitochondrial damage inside the cancer cells (Fig. 4**)** [75], [76], [77]. Additionally, their protective effect against bacterial, fungal, and viral infections could be very desirable during chemo- and radiotherapies due to the decreased immunological resistance of cancer patients [78].

Recently, Zangeneh et al. employed *Spinacia oleracea* leaf, commonly known as spinach, to produce AgNPs that were tested as antileukemic agents in an animal model of AML, while comparing it with anticancer activity of doxorubicin (DOX). *Spinacia oleracea* extracts contain more than 13 different types of flavonoids, which could act as anticancer and antioxidants agents; hence some synergy with the AgNPs was expected upon its use as reducing/capping agent [79]. Like DOX, green synthesized NPs showed high *in vitro* anticancer activity against different cancer cells, while (and unlike DOX) biogenic NPs had no toxicity effect against normal cells. In the animal models, AgNPs significantly reduced the concentration of pro‐inflammatory cytokines, the counts of different types of white blood cells (including neutrophils, eosinophil, monocyte, and basophil); At the same time, it led to an increase in body weight, lymphocyte, platelet, red blood cell parameters, and the anti‐inflammatory cytokines in comparison to the untreated mice. The obtained results confirmed that the *S. oleracea* leaf extract could promote the biomedical features of AgNPs. The fabricated NPs showed high-performance antioxidant, anti‐acute, and anticancer capabilities against myeloid leukemia [80].

*Melissa officinalis*, commonly known as lemon balm, was employed to produce AgNPs under the same conditions, revealing antioxidant and anticancer effects against myeloid leukemia in mice models compared to the chemotherapeutic drug (mitoxantrone). The quantitative results of real‐time PCR tests showed a significant increase in the expression level of S1PR1 and S1PR5 mRNAs after treatment with AgNPs and mitoxantrone, hence allowing a potential venue for synergy of both elements. Besides, AgNPs showed similar hematological properties to mitoxantrone, so the platelet, lymphocyte, and RBC parameters were significantly enhanced. Comparable immunological parameters to mitoxantrone were also recorded, as the anti‐inflammatory and pro‐inflammatory cytokines were reduced compared to the untreated mice [81].

In a different approach, AgNPs were fabricated by utilizing the phytochemicals isolated from *Boswellia dalzielii* stem bark, a common tree found in Africa. The as-prepared AgNPs had sizes between 12 and 101 nm and contained plant phytochemicals, such as aromatic compounds, ethers, and alkynes on their surface as coating layer [82]. The AgNPs showed half minimal inhibitory concentrations (IC50s) effect at 49.5 μg/ml and 13.25 μg/ml after 48 and 72 h, respectively, against Kasumi 1 leukemic cell line and induced cell cycle arrest in the cells at the phase S (5% increase) and phase G2/M (3% increase) [83].

Ahmeda et al. compared the anti-leukemic activity of phytosynthesized spherical AgNPs with a mean diameter of 20 nm and cationic silver nitrate against four types of acute T cell leukemia cell lines (Jurkat Clone E6–1, J.CaM1.6, J45.01, and J.RT3‐T3.5). The anticancer activity was performed using an MTT assay. After 48 h of treatment, the IC50 values of AgNPs were found to be 329, 284, 226, and 270 µg/mL against J45.01, Jurkat Clone E6–1, J.CaM1.6, and J.RT3‐T3.5 cell lines, respectively. Significantly, no IC50 value was found for silver nitrate (even at concentrations more than 1000 µg/mL) against all of the mentioned cell lines. Additionally, to make sure that the anti-leukemic activity of AgNPs is not attributed to the plant extract that was utilized for fabrication of AgNPs, the authors evaluated the cytotoxicity of the aqueous Glycyrrhiza glabra L leaf extract and reported the IC50 values of 604, 467, 445, and 438 µg/mL against J45.01, Jurkat Clone E6–1, J.CaM1.6, and J.RT3‐T3.5 cell lines, respectively [84].

More recently, AgNPs were successfully produced and purified from the *Achillea millefolium* extracts, also known as yarrow, which are simultaneously used as a reducing and capping agent. The green synthesized AgNPs were spherical with an average size of 22.4 ± 7.4 nm, which showed cytotoxicity effects against precursor T-cells of ALL (MOLT‐4) with IC50 of about 0.011 µm in comparison to 1.7 µm for cisplatin that confirmed their strong leukemogenic potential [85].

Pattanayak et al. fabricated green AgNP via utilizing *Butea monosperma* bark extract with a mean size of about 35 nm **(**Figs. 5**A and** 5**B**). They showed significant anti-leukemic activity of herbal-mediated fabricated AgNPs against human myeloid leukemia cells (KG-1A) with an IC50 value of 11.47 µg/mL. The cellular morphology of AgNPs treated cells was measured using the Acridine orange (AO)–ethidium bromide (EtBr) double staining technique. The fluorescence images of stained cells with the AO-EtBr double staining technique **(**Fig. 5**C (a and b)**) represent the round and green nuclei in untreated cells due to viable cells with intact DNA and nuclei. Moreover, in AgNPs treated cells, several green-colored nuclei indicated early apoptotic cells with fragmented DNA. Remarkably, it is noted that for the case of late apoptosis and necrosis, the cells are stained in orange and red color, respectively. Furthermore, the DAPI staining technique revealed chromatin condensation and fragmentation as a hallmark of apoptosis in AgNPs treated KG-1A cells **(**Fig. 5**C (c and d))** [86].

In a different study, the extract of the night-flowering jasmine (*Nyctanthes arbor-tristis*) was employed as a stabilizing and reducing agent for the fabrication of AgNPs in a handy, cost-effective, and reproducible method. The dried leaf, fruit, and stems of *Nyctanthes arbortristis* have been previously investigated as anticancer agents on MDA-MB 231 breast cancer cell lines, hence becoming a suitable agent for synthesizing the AgNPs to find a boost of this indication [87]. Flavonoids, glycosides, and phenols, which were presented in the leaves extract, were the main phytochemicals responsible for the nano-transformation. The fabricated AgNPs showed dose-dependent cytotoxicity against THP-1 human leukemia cell lines, which was resulted from the production of high amounts of ROS inside the cells that led to damaging cellular organelles and, finally cell death [88].

Alternatively, to detect the effect of different components of herbal extract on the anticancer properties of AgNPs, four different types of phenylpropanoids (including ferulic acid (FA), sinapic acid (SA), caffeic acid (CA), and chlorogenic acid (CHA)) were isolated from the water extract of *Suaeda maritima*, and their anticancer effects were evaluated against K562 cells (human myeloid leukemia). This study revealed that the AgNPs synthesized from FA and CHA have significant anticancer activity against leukemia even in lower doses. They also had no significant cytotoxicity effect against normal cell lines [89].

Maity et al. reported the significant anti-leukemic activity of biosurfactant-mediated synthesized AgNPs against KG-1A and K-562 cell lines with IC50 values at 47.94 and 49.63 µg/mL, respectively. Fig. 6**A** represents the SEM and TEM images of the cellular membrane of KG-1A and K-562 cells after exposure to the biosynthesized Ag compared to their untreated cells, indicating the altering cell membrane integrity through AgNPs treated cells. In other words, these NPs could induce their cytotoxic effect via a significant increase in the amounts of ROS through affecting the cellular redox balance, changing the mitochondrial membrane potential, generating apoptosis via destroying the nucleus structures (Figs. 6**B and** 6**C)**) [90].

Adding to the evidence of anticancer activities of biogenic AgNPs, Govindaraju and colleagues employed *Sargassum Vulgare,* an algae species, to prepare stabilized AgNPs under ambient conditions. The algae extracts have been previously reported as useful anticancer agents with high cytotoxic activity against Jurkat human leukemic cell lines [91]. The resulting structure exhibits a concentration-dependent cytotoxic effect against human myeloblastic leukemic HL60 cell lines. The authors explored the mechanism of death through a DNA fragmentation study, confirming apoptosis in leukemic cells. In addition, the study provides more evidence of ROS generation and DNA damage of HL60 cells post-endocytosis [92].

Green synthesized AgNPs were also used to induce autophagy in leukemic cancer cells and inhibit the NLR family pyrin domain containing 3 (NLRP3) inflammasome. In this research, *Annona muricata* peel extract was acted as reducing agent for the preparation of AgNPs with an average size of about 17 nm, which could improve the autophagy via controlling the interleukin 1β (IL-1β) pathway through two different methods; 1) controlling the amounts of IL-1β, caspase-1, and adaptor protein apoptosis-associated speck-like protein containing a CARD (ASC), and 2) degradation of NLRP3 by lysosomal enzyme. It could also inhibit the cell proliferation of THP-1 leukemia cancer cells and induce apoptosis via stimulation of p53 protein and generation of mitochondrial damages (Fig. 7) [93].

Table 1 summarizes the advancement in green synthesis of AgNPs and their approaches for leukemia therapy.

**Table 1:** Summary of Green-synthesized AgNPs structures with anti-leukemic applications.

Biological source	Size (nm)	Leukemia cell line	Dose	IC50 value	Ref
*Spinacia oleracea*	20 - 40	32D-FLT3-ITD&Human HL-60/vcr Murine C1498	0–1000 µg/mL	214 µg/mL 238 µg/mL 217 µg/mL	[94]
*Glycyrrhiza glabra*	20	J45.01 Jurkat Clone E6–1 J.CaM1.6, J.RT3.T3.5	0–1000 µg/mL	329 µg/mL 284 µg/mL 226 µg/mL 270 µg/mL	[95]
*Melissa officinalis*	5 – 30	32D-FLT3-ITD Human HL-60/vcr Murine C1498	0–1000 µg/mL	325 µg/mL 272 µg/mL 188 µg/mL	[96]
*Boswellia dalzielii*	12 – 101	Kasumi 1	0–70 μg/ml	49.5 µg/mL (24 h) 13 µg/mL (72 h)	[97]
*A. millefolium*	22.4 ± 7.4	MOLT-4	0.004–0.1 µM	0.011 uM	[98]
*Nyctanthes arbortristis*	12 - 22	THP-1	5–50 µg/ml	30 – 40 µg/mL	[99]
*Sargassum Vulgare*	7 - 12	HL60	0.2–100 µg/ml	2.84 µg/mL	[100]
*Azadirachta indica*	20–50 nm	human acute lymphocytic leukemia (ALL)	15.6–250 µg/mL	Around 40% cell inhibition was found at 250 µg/mL	[101]
*Calotropis gigantea*	Average: 2.338 nm	Jurkat	0.5–10 µg/mL	11.99 µg/mL	[102]
*Nathophodytes foetida*	No data	K-562	1–30 µg/mL	10 µg/mL	[103]
*Nothapodytes foetida*	10–50 nm	K-562	1–30 µg/mL	6.17 µg/mL	[104]

* Abbreviations: murine AML cell line from C57BL/6 mouse (C1498); Human leukemia cells HL60 (HL60); murine myeloid progenitor 32D cells that ectopically express human FLT3-ITD (32D- FLT3-ITD); T lymphoblast ALL derived cells MOLT-4 (MOLT-4); Human myeloblast AML derived cells (Kasumi 1); human bone marrow lymphoblast CML derived cells (K562); human AML monocytes (THP-1); derivative mutant of the Jurkat leukemia cell line (J .RT3 -T3.5); immortalized line of human T lymphocyte cells (Jurkat); derivative mutant of Jurkat (J.CaM1.6).

### 2.2 Biogenic gold nanoparticles (AuNPs) for leukemia therapy

AuNPs have been introduced as promising agents for cancer therapy that could be used as drug carrier, photothermal agent, contrast agent, and radiosensitiser [105, 106]. The cytotoxic effect of AuNPs has resulted from the physicochemical interactions between gold atoms and the functional groups of different intracellular proteins, as well as the phosphate groups and nitrogen bases of DNAs [105].

The biogenic AuNPs could increase the accumulation of treated cells in the sub-G1 phase and induce apoptosis in these cells via activation of different mechanisms like chromatin condensation, membrane blebbing, and cell shrinkage (Fig. 8) [107]. Several research groups have been used green synthesized AuNPs to manage Leukemia. For instance, the aqueous extracts of the brown seaweed *Sargassum glaucescens* were used to synthesize AuNPs, and then the anticancer effect of the NPs against four different cancer cell types (among them was CEM-ss as leukemia cancer cell) was evaluated. NPs had a mean size of about 3.65 nm with dose-dependent anticancer activity against all cancer cells, while no toxicity was seen against the normal cell. In the case of leukemia cells, it showed IC50 at the concentration of 10.32 µg/ml. Moreover, they confirmed that the high toxicity effect of the biosynthesized NPs resulted from Au since in the case of extract, much lower toxicity has occurred [108].

Zangeneh et al. assessed the use of *Hibiscus sabdariffa*, also known as roselle, for the biosynthesis of AuNPs. The presence of high amounts of polyphenolic agents in the crude and pure compounds of extract provide excellent features for them, including selective cytotoxicity, apoptosis, cell cycle arrest, anti-metastasis, and autophagy effects against several types of human cancer cells [109]. Results of this study confirmed the therapeutic activity of the nanosystem against acute myeloid leukemia rodent model. The *in vitro* results showed that AuNPs decreased the viability of the cells in a dose‐dependently manner against three different cancer cell lines (Murine C1498, Human HL‐60/vcr, and 32D‐FLT3‐ITD cell lines) without any cytotoxicity on Human umbilical vein endothelial cells (HUVEC) cell line. The results of *in vivo* experiments revealed that mice treated with AuNPs showed comparable effects to DOX via reducing the pro‐inflammatory cytokines and the total white blood cells, blast, monocyte, neutrophil, eosinophil, and basophil counts, and also enhancement of the anti‐inflammatory cytokines and the platelet, lymphocyte, and RBC parameters in comparison to the control. These results revealed the potential use of these plant-synthesized AuNPs as potential antileukemic agents [110].

In a similar approach, *Camellia sinensis*, a species of evergreen shrubs, were employed to produce AuNPs. Catechins, molecules present in high content in the extracts, contain four main components: epicatechin, epicatechin gallate, epigallocatechin, and epigallocatechin gallate. They are also known as one of the strongest anti-inflammatory and anticancer agents in all plant extracts. Indeed, the catechins of green tea have been shown to have a significant anticancer effect against several types of cancer, from lung and breast cancer to liver cancer, esophageal cancer, prostate cancer, and stomach cancer [111]. Therefore, it could be expected that the combined use of these components with AuNPs could lead to a synergetic anticancer effect [112]. The anticancer activity of the synthesized nanostructure was compared with DOX in mice animal models with AML. FTIR results confirmed the reducing property of the antioxidant compounds of the extract, which were used for the fabrication of AuNPs. The fabricated NPs had low cell viability and dose‐dependent toxicity against different types of leukemia cell lines. Similar to previous results, these NPs enhanced the amounts of anti‐inflammatory cytokines, the lymphocyte, platelet, and RBC parameters and reduced the weight and volume of liver and spleen, the pro‐inflammatory cytokines, and the total WBC, compared to the untreated mice [113].

The anticancer activity of biosynthesized AuNPs against leukemia was also compared with that of mitoxantrone. In detail, AuNPs with mean size of about 25 nm were synthesized using extract of aqueous *Lens culinaris* (L. *culinaris*) seed. Then different features of these NPs were compared with mitoxantrone. It was confirmed that both had the same *in vitro* antioxidant and anticancer activities against leukemia cancer cells and could decrease the volume and weight of spleen, liver, total WBC, and pro-inflammatory cytokines *in vivo.* They could also increase some other parameters like the expression of sphingosine1-phosphate receptor-5 and sphingosine-1-phosphate receptor-1 mRNAs and the anti-inflammatory cytokines *in vivo* model. These results revealed that these biosynthesized AuNPs could be either used in combination with chemotherapeutic drugs or even instead of them (Fig. 9) [114].

Justus et al. compared the anti-leukemic activity of the AuNPs and AgNPs, which were fabricated using the aqueous extract of *Lavandula dentata* L, against K-562 cells using MTT assay. After 72 h of treatment, 250 µL of AuNPs stock (0.1 mM) induced 76.2% cell inhibition, whereas AgNPs induced 36.1% reduction in cell viability [115].

Providing more evidence, Hemmati and colleagues studied the ability of aqueous extract of *Thymus vulgaris* (or thyme) for the biosynthesis of AuNPs. *T. vulgaris* extracts have been shown anticancer effects in human colorectal cancer cells via increasing the apoptotic cell death via elevating the activity of caspase3/7 and inhibiting the metastasis and invasive capabilities of HCT [116] cells108108. After an *in vitro* study with leukemic cell lines, the results of the mice model showed that the expression of sphingosine‐1‐phosphate receptor‐1 and sphingosine‐1‐phosphate receptor‐5 mRNAs in lymphocytes of leukemic mice were significantly increased after AuNPs and DOX treatment [117].

Table 2 summarizes the advancement in green synthesis of AuNPs and their approaches to leukemia therapies.

**Table 2:** Summary of Green-synthesized AuNPs structures with anti-leukemic applications.

Biological source	Size (nm)	*In vitro* cell lines	Dose	IC50 value	Ref.
*Hibiscus sabdariffa*	15–45	C1498 HL‐60/vcr 32D‐FLT3‐ITD	0–1000 µg/ml	185 µg/mL 189 µg/mL 309 µg/mL	[118]
*Camellia sinensis*	20–30	C1498 HL‐60/vcr 32D‐FLT3‐ITD	0–1000 µg/ml	158 µg/mL 224 µg/mL 258 µg/mL	[119]
*Boswellia serrata*	15–30	C1498 HL‐60/vcr 32D‐FLT3‐ITD	0–1000 µg/ml	219 µg/mL 329 µg/mL 320 µg/mL	[120]
*Lens culinaris*	10–40	C1498 HL‐60/vcr 32D‐FLT3‐ITD	0–1000 µg/ml	212 µg/mL 246 µg/mL 367 µg/mL	[121]
*Thymus vulgaris*	10–30	C1498 HL‐60/vcr 32D‐FLT3‐ITD	0–1000 µg/ml	186 µg/mL 218 µg/mL 336 µg/mL	[122]
*Cannabis sativa*	18.6	MOLT-3 TALL-104 J .RT3 -T3.5	0–1000 µg/ml	329 µg/mL 381 µg/mL 502 µg/mL	[123]
*Verbena officinalis*	35	Jurkat	0.1–100 µmol	1,08 µmol (24 h) 0.65 µmol (48 h) 0.39 µmol (72 h)	[124]
*Thymus vulgaris*	10–30	Murine C1498 Human HL‐60/vcr 32D‐FLT3‐ITD	1–1000 µg/mL	186 µg/mL 218 µg/mL 336 µg/mL	[122]
*Boswellia serrata*	15–30	Murine C1498 Human HL‐60/vcr 32D‐FLT3‐ITD	1–1000 µg/mL	219 µg/mL 329 µg/mL 320 µg/mL	[125]
*Abelmoschus esculentus* L.	4–32	Jurkat K-562	1–50 µg/mL	8.17 µg/mL ∼50 µg/mL	[126]
*Dracocephalum kotschyi*	11	K-562	62.5–500 µg/mL	196.32 µg/mL	[127]
*Sargassum glaucescens*	3.65±1.69	CEM-ss	0–100 µg/mL	10.32±1.5 µg/mL	[108]
Diallyl disulfide (a bioactive garlic compound)	70–77	U937 K-562	5–20 µg/mL	14.164±0.62 µM 15.82±0.798 µM	[128]

* Abbreviations: murine AML cell line from C57BL/6 mouse (C1498); Human leukemia cells HL60 (HL60); murine myeloid progenitor 32D cells that ectopically express human FLT3-ITD (32D- FLT3-ITD); T lymphoblast AML derived cells MOLT-3 (MOLT-3); peripheral blood T-ALL cells (T-ALL); derivative mutant of the Jurkat leukemia cell line (J .RT3 -T3.5); immortalized line of human T lymphocyte cells (Jurkat).

## 3 Challenges and prospects

Although AuNPs and AgNPs are promising approaches for their use in leukemia theragnostic, some challenges still need to be overcome before their use in clinical trials. Even though toxicity issues seem to have improved compared to chemically synthesized nanomaterials, there is still a huge concern regarding the long-term effect of AuNPs and AgNPs, which is related to the metallic nature of these NPs. Moreover, parameters such as size or synthesis conditions (pH, raw material, pressure, or drug concentration) can have a vital role in their therapeutic properties and biodistribution [129, 130]. Therefore, there is a need for an adequate evaluation of their efficacy, accumulation in the body, and possible immune reaction [131].

Biocompatibility and tissue penetration concerns are especially important in photothermal therapy, making it difficult to use any kind of NPs as photothermal agents [132]. For example, Aspargine-laminated AuNPs synthesized using *Camilla sinensis* extract were successfully used to potentiate the heat exchange when treating T-cell leukemia cells. The particles could perform up to two times higher than other mechanisms inducing necrosis and apoptosis to cells [133].

In addition, there are other hurdles to introduce AuNPs and AgNPs in leukemia theranostics, particularly those are related to the proper uptake, diffusion, and penetration of the NPs into the cells to ensure high effectivity of the NPs [134, 135]. Unfortunately, most of these mechanisms have not been extensively reported in the literature for the afore-mentioned nanostructures. As seen in Ma et al., AuNPs were uptake in a size-dependent manner by the cells through endocytosis. Once the particles penetrated, they accumulated into the lysosomes causing lysosomal degradation [136].

Another main issue in the production of nanomaterials using green methodologies is the homogeneity of the raw material, which is especially significant on plants. Living organisms within the same species do not present the exact same composition, which might affect NPs synthesis and final properties of the materials giving problems on robustness and reproducibility of the materials [137, 138]. Furthermore, the processes of reduction to generate these nanomaterials, generally, cannot be controlled. Additionally, the chemical compounds within the living organisms that are in charge of the reduction process are often hard to identify [139].

Despite the promise of anticancer activities presented throughout the review, these claims must be carefully taken into consideration, as most of them do not have clinical data to back up the statements. Nonetheless, a handful of articles and reviews have published pre-clinical data and animal studies associated with the anticancer and antileukemic claims of both NPs and plant extracts. With these pre-clinical data, and a sound approach to addressing the concerns regarding biodistribution, reproducibility, and safety, clinical validations remain the next steps in advancing green nanostructures as therapeutic modalities into clinical translation. In addition, the development of therapeutic green synthesized nanostructures is not meant to displace or substitute current treatments but to provide a complementary approach to improve the prospects of leukemia in society.

## 4 Conclusion

The biogenic AgNPs and AuNPs represent the frontier of a novel class of therapeutic: green-synthesized nanostructures. This review detailed some current strategies to produce AgNPs and AuNPs from natural organisms and evaluated their cytotoxic activities against leukemic targets. These findings indicate comparable anticancer properties to some of the existing therapeutic agents and satisfactory safety compatibility to human cells. Therefore, AuNPs and AgNPs are introduced as powerful alternatives to current approaches to treat leukemia. Nonetheless, there is still a series of challenges that need to be overcome in terms of greater understanding of toxicity, biodistribution, and process quality for biogenic NPs to achieve clinical translation.
